# Excision Repair Cross-Complementation Group 6 Gene Polymorphism Is Associated with the Response to FOLFIRINOX Chemotherapy in Asian Patients with Pancreatic Cancer

**DOI:** 10.3390/cancers13061196

**Published:** 2021-03-10

**Authors:** Young Hoon Choi, Younggyun Lim, Ji Kon Ryu, Woo Hyun Paik, Sang Hyub Lee, Yong-Tae Kim, Ju Han Kim

**Affiliations:** 1 Department of Internal Medicine, College of Medicine, The Catholic University of Korea, Seoul 06591, Korea; crzyzs@naver.com; 2 Department of Internal Medicine and Liver Research Institute, Seoul National University Hospital, Seoul National University College of Medicine, Seoul 03080, Korea; iatrus@hanmail.net (W.H.P.); gidoctor@snuh.org (S.H.L.); yongtkim@snu.ac.kr (Y.-T.K.); 3 Seoul National University Biomedical Informatics (SNUBI), Division of Biomedical Informatics, Seoul National University College of Medicine, Seoul 03080, Korea; csyj2002@snu.ac.kr (Y.L.); juhan@snu.ac.kr (J.H.K.)

**Keywords:** pancreatic cancer, FOLFIRINOX, DNA repair, *ERCC6*, progression-free survival

## Abstract

**Simple Summary:**

FOLFIRINOX is a platinum-based chemotherapy regimen for patients with pancreatic cancer and is known to be more effective in the presence of the BRCA mutation, one of the DNA damage repair (DDR) gene mutations. However, BRCA mutations are less common in pancreatic cancer patients, accounting for only about 5% of cases worldwide, and are known to be even rarer in Asians. Therefore, this study aimed to uncover new genetic variants of DDR genes related to the response of FOLFIRINOX by analyzing variants of DDR genes using whole exome sequencing. Multivariable Cox regression analysis adjusted for clinical variables showed that a single nucleotide polymorphism (SNP) of the *ERCC6* gene is an independent predictor for progression-free survival. If validated, the *ERCC6* SNP found in this study could be used as a biomarker to predict responses to FOLFIRINOX.

**Abstract:**

FOLFIRINOX is currently one of the standard chemotherapy regimens for pancreatic cancer patients, but little is known about the factors that can predict a response to it. We performed a study to discover novel DNA damage repair (DDR) gene variants associated with the response to FOLFIRINOX chemotherapy in patients with pancreatic cancer. We queried a cohort of pancreatic cancer patients who received FOLFIRINOX chemotherapy as the first treatment and who had tissue obtained through an endoscopic ultrasound-guided biopsy that was suitable for DNA sequencing. We explored variants of 148 DDR genes based on whole exome sequencing and performed multivariate Cox regression to find genetic variants associated with progression-free survival (PFS). Overall, 103 patients were included. Among 2384 variants of 141 DDR genes, 612 non-synonymous variants of 123 genes were selected for Cox regression analysis. The multivariate Cox model showed that rs2228528 in *ERCC6* was significantly associated with improved PFS (hazard ratio 0.54, *p* = 0.001). The median PFS was significantly longer in patients with rs2228528 genotype AA vs. genotype GA and GG (23.5 vs. 16.2 and 8.6 months; log-rank *p* < 0.001). This study suggests that rs2228528 in *ERCC6* could be a potential predictor of response to FOLFIRINOX chemotherapy in patients with pancreatic cancer.

## 1. Introduction

Pancreatic cancer is a lethal disease with a 5-year survival rate of 9% [1]. One of the reasons for this poor prognosis is that 85–90% of patients are diagnosed at an advanced stage where surgical resection is not possible [1,2]. Therefore, a large number of pancreatic cancer patients receive chemotherapy as an initial treatment, and the two preferred chemotherapy regimens today are FOLFIRINOX and Gemcitabine plus albumin-bound paclitaxel [3,4,5]. Predicting which of these two chemotherapy regimens will be more effective for each patient helps determine the best first-line chemotherapy. One known factor in this regard is that patients with mutations in DNA damage repair (DDR) genes respond well to FOLFIRINOX. The FOLFIRINOX regimen consists of four drugs: Oxaliplatin, leucovorin, irinotecan, and 5-fluorouracil [4]. Of these, oxaliplatin is a platinum-based anticancer drug that uses gene disruption as a mechanism [6]. In patients with mutations in the DDR gene, the addition of gene disruption by platinum-based chemotherapy leads to cancer cell death by synthetic lethality, resulting in a better response to platinum-based chemotherapy [7,8]. In particular, the BRCA mutation is the best-known DDR gene mutation, and the National Comprehensive Cancer Network guideline recommends the use of FOLFIRINOX in the presence of the BRCA mutation [3]. However, BRCA mutations are not very common in pancreatic cancer patients, occurring at a frequency of about 5% of cases worldwide, and there are reports that the frequency of BRCA mutations is even lower in Asians, at a frequency of less than 1% [9,10]. Therefore, further research is needed regarding whether other variants of DDR genes are associated with a good response to FOLFIRINOX, especially in Asians. Although a few studies have reported genes associated with FOLFIRINOX chemotherapy responses, those studies were based on targeted gene sequencing and were therefore limited to the analysis of only a few DDR genes [11,12]. Thus, in this study, we aimed to find novel variants of DDR genes that could predict the response to FOLFIRINOX chemotherapy based on whole exome sequencing analysis.

## 2. Materials and Methods

### 2.1. Patients and Data Collection

Patients were selected from the prospectively collected pancreatic cancer database of the Seoul National University Hospital between May 2017 and May 2019. Patients with the following criteria were included: (i) Histologic diagnosis of pancreatic ductal adenocarcinoma from tissue obtained through endoscopic ultrasound (EUS)-guided fine needle biopsy (EUS-FNB); (ii) locally advanced or metastatic pancreatic cancer; (iii) chemotherapy with FOLFIRINOX regimen as the first line; (iv) available tissue obtained through EUS-FNB for DNA sequencing. Patients who discontinued FOLFIRINOX chemotherapy despite no disease progression in computed tomography (CT) scan (due to patient’s refusal, poor performance status, or adverse effects of the FOLFIRINOX regimen) or who were lost to follow-up or transferred out were excluded.

Baseline demographic and clinical data, including age, sex, body mass index (BMI), Eastern Cooperative Oncology Group (ECOG) performance status, primary tumor location, primary tumor size, node involvement, distant metastasis, tumor-node-metastasis stage by the 8th American Joint Committee on Cancer (AJCC) classification [13], baseline serum carbohydrate antigen 19-9 (CA 19-9) level, best response to FOLFIRINOX chemotherapy, surgical resection after FOLFIRINOX chemotherapy, and survival were obtained from medical records. Assessment of chemotherapy response was performed by CT scan every three or four cycles of chemotherapy according to the Response Evaluation Criteria in Solid Tumor version 1.1 [14]. Written informed consent was obtained from all patients. The study was conducted in accordance with the Declaration of Helsinki and was approved by the Institutional Review Board of Seoul National University Hospital.

### 2.2. Sample Acquisition through EUS-FNB and DNA Extraction

EUS-FNB was performed by one of four expert endoscopists with experience of >1000 cases for pancreatic diseases. Patients underwent conventional EUS-FNB using a linear-array echoendoscope (GF-UCT 240, GF-UCT 260; Olympus Optical Co., Tokyo, Japan) and a 19- or 22-gauge needle (EZ Shot 3; Olympus Medical, Tokyo, Japan, Acquire; Boston Scientific, MA, USA). For histological diagnosis, tissue was obtained through at least two needle passes, and if there was no visible core tissue, up to two more passes were performed. Afterwards, samples for biobanking were obtained through 1–2 needle passes to obtain visible core tissue. These specimens for biobanking were placed in a cryotube and then immediately frozen in liquid nitrogen and stored in a deep freezer at −80 °C until DNA extraction. The HiGene Genomic DNA Prep Kit (GD141-100, BIOFACT, Daejeon, Korea) was used for DNA extraction.

### 2.3. Whole Exome Sequencing and Variant Calling

Exome sequencing was conducted using the Ion AmpliSeq Exome panel (A29854, Thermo Scientific, MA, USA) to screen the entire genome’s coding sequence regions. Sequencing libraries were prepared using the Ion Ampliseq Library Kit Plus (A29854, Thermo Scientific, MA, USA). Libraries were quantified using the Agilent 2100 Bioanalyzer (Agilent, CA, USA) and then diluted to about 100 pM. Subsequently, 50.0 μL of the barcoded libraries were combined into sets of two barcodes. The combined libraries were sequenced using the Ion S5XL platform with 540 Chip (A27766, Thermo Scientific). Torrent Suite Software v5.0.2 was employed for generating mapped reads to the human reference genome build (hg19) with germ-line and low-stringency settings. Single-nucleotide polymorphism (SNP) variants and short insertions/deletions (INDELs) were identified using the Genome Analysis Toolkit 2.8-1 UnifiedGenotyper [15] and Torrent Variant Caller plugin v1.0.0. Raw reads were aligned to the reference human genome (hg19/GRCh37). We manually reviewed the sequence alignment of whole variants by IGV 2.8.9 [16] to exclude false-positive variant calls. All called variants were annotated using ANNOVAR [17]. We set up the DDR genes to be analyzed with reference to the previous study [18] (Supplementary Table S1), and only non-synonymous variants of these DDR genes that have the potential to affect gene function were included in the further analysis.

### 2.4. Statistical Analysis

Continuous data are shown as median and interquartile ranges, whereas categorical data are shown as number and percent. Progression-free survival (PFS) was defined as the interval between the start date of FOLFIRINOX and the date of disease progression or death. PFS was assessed using the Kaplan–Meier method and the log-rank test. A *p*-value < 0.05 was considered significant. Multivariate Cox proportional hazard regressions adjusted for clinical variables were performed to evaluate the association between PFS and each of the genetic variants of DDR genes. The adjusted clinical variables were age, sex, tumor–node–metastasis stage, tumor location, body mass index, serum CA 19-9, and surgical resection after chemotherapy. Benjamini–Hochberg multiple testing correction was applied to estimate the false discovery rate (FDR). An FDR-adjusted *p*-value < 0.1 was considered statistically significant. All statistical analyses were performed using SPSS version 24.0 (IBM Corp., Armonk, NY, USA), MedCalc Statistical Software version 19.6.1 (MedCalc Software Ltd., Ostend, Belgium), and R 3.6.3 (the R Foundation for Statistical Computing, Vienna, Austria).

## 3. Results

### 3.1. Patient Characteristics

**Figure 1:**
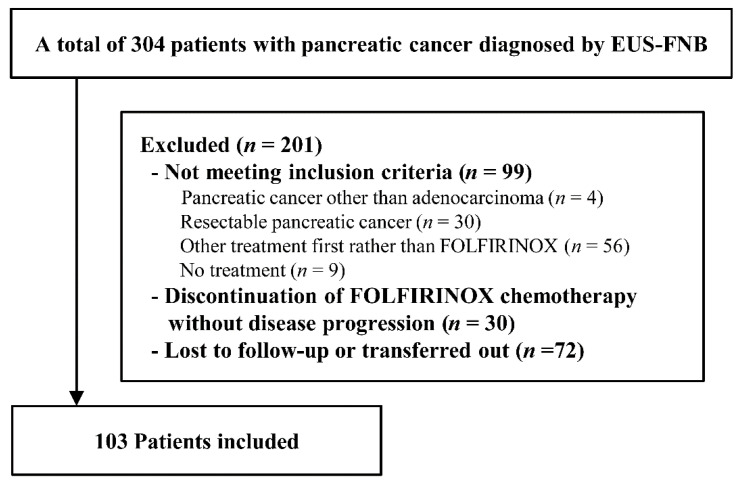
Flow chart of patient enrollment. The flowchart shows the patient exclusion criteria and the final number of patients included in the study.

During the study period, 304 patients were diagnosed with pancreatic cancer through endoscopic ultrasound EUS-FNB. Of these patients, we excluded patients who did not meet the inclusion criteria (*n* = 99), discontinued FOLFIRINOX treatment without disease progression (*n* = 30), or were lost to follow-up or transferred out (*n* = 72). A total of 103 patients were included in this study (Figure 1).

Baseline characteristics of patients are shown in Table 1. The median age of the study patients was 64 years. All patients had an ECOG performance status of 0 or 1. Metastatic pancreatic cancer was 35.9%. In response to FOLFIRINOX treatment, 32% of patients achieved partial response, 55.3% of patients achieved stable disease, and 12.6% of patients had progressive disease. Surgical resection after FOLFIRINOX treatment was performed in 39.8% of patients.

### 3.2. Genetic Variants of DDR Genes Predicting PFS

**Figure 2:**
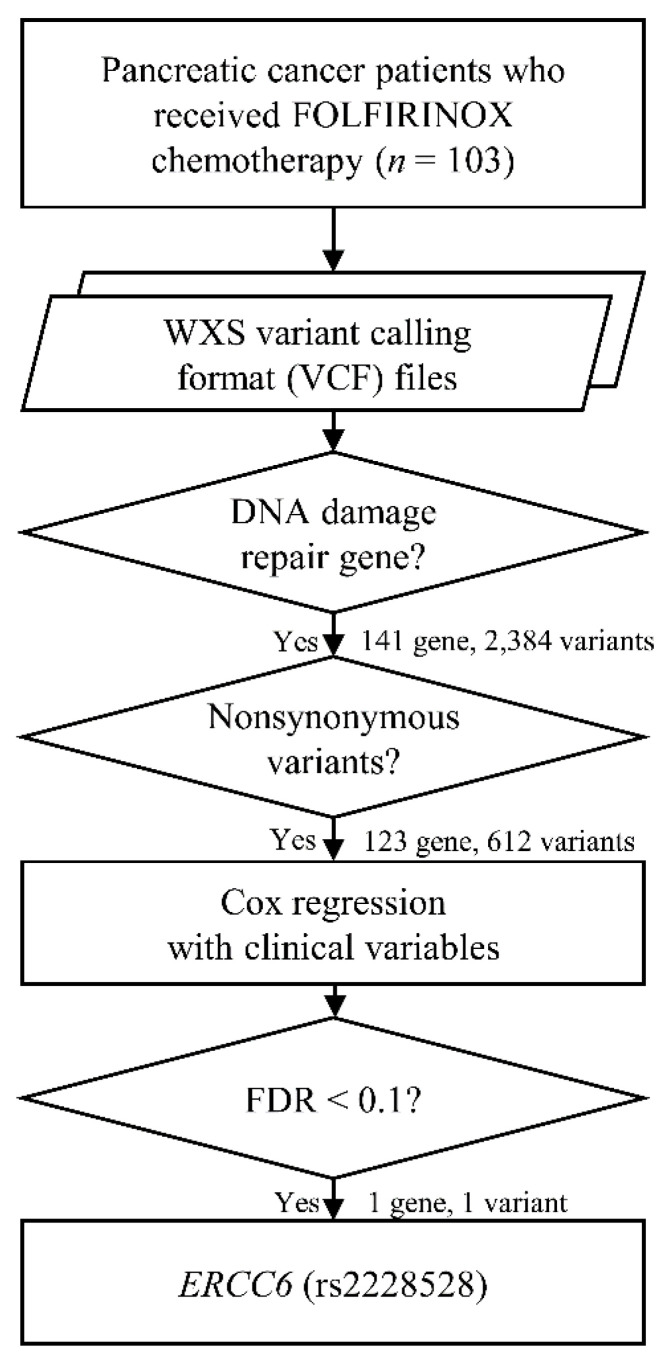
Schematic diagram of genetic variant analysis. The schematic diagram shows how genetic variants were selected and analyzed from whole exome sequencing data. Abbreviation: WXS, whole exome sequencing.

Of the 148 DDR genes we analyzed, a total of 2384 variants were found in 141 genes. Among these variants, there were 612 non-synonymous variants in 123 genes. Multivariate Cox regression adjusted for clinical factors showed that out of 612 non-synonymous variants, only rs2228528 in the gene ERCC6 was a genetic variant significantly associated with PFS (hazard ratio (HR) 0.54, *p* = 0.001, FDR adjusted *p* = 0.08) (Figure 2).

### 3.3. PFS Analysis According to rs2228528 in ERCC6

**Figure 3:**
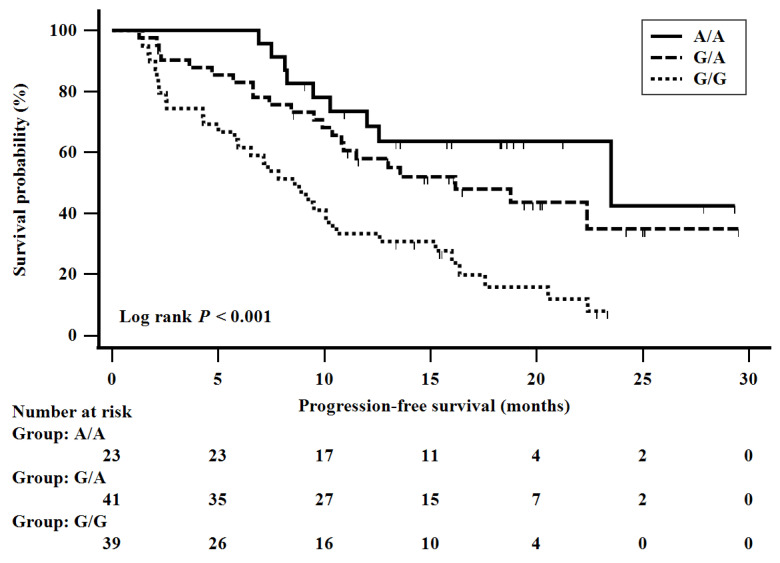
Progression-free survival according to genotype of rs2228528 in ERCC6. Patients were divided according to rs2228528 genotypes: AA (*n* = 23), GA (*n* = 41), and GG (*n* = 39), and assessed for progression-free survival using the Kaplan–Meier method and the log-rank test.

There are three rs2228528 genotypes in ERCC6: GG, GA, and AA. The number of patients for each genotype was GG (*n* = 39), GA (*n* = 41), and AA (*n* = 23). The median PFS was 8.6 months (95% confidence interval (CI), 5.8–10.6) for the GG genotype carriers, 16.2 months (95% CI, 10.8–22.4) for the GA genotype carriers, and 23.5 months (95% CI, 12.0–23.5) for the AA genotype carriers (Figure 3).

### 3.4. Factors Predicting PFS

Multivariate Cox model for PFS showed that rs2228528 genotype with the A allele in ERCC6 (HR 0.54; 95% CI, 0.37–0.78, *p =* 0.001) and surgical resection after FOLFIRINOX chemotherapy (HR 0.27; 95% CI, 0.14–0.52, *p <* 0.001) were independent predictors for better PFS, whereas metastasis stage M1 (HR 2.21; 95% CI, 1.16–4.21, *p =* 0.016) was an independent predictor for poor PFS (Table 2).

## 4. Discussion

In this study, we demonstrated that the A alleles of rs2228528 in *ERCC6* were significantly associated with longer PFS in pancreatic cancer patients who received FOLFIRINOX chemotherapy. This suggests that the allele A carriers of rs2228528 in *ERCC6* had a better response to FOLFIRINOX than the allele G carriers. To the best of our knowledge, this is the first study to report that an SNP of *ERCC6* is related to the response to FOLFIRINOX chemotherapy. *ERCC6* plays important roles in the nucleotide excision repair (NER) pathway, one of the pathways in the DDR response [19,20]. The NER pathway repairs DNA damage through steps involving recognition of a DNA damage lesion, unwinding DNA, making incisions around the DNA lesion, and resynthesis and ligation of DNA [21]. DNA damage recognition by the NER pathway consists of two arms: The global genome repair (GGR) branch that recognizes non-transcribed lesions and the transcription-coupled repair (TCR) branch that recognizes transcribed lesions [22]. *ERCC6* is an important component of TCR. If DNA damage in a transcribed gene cannot be repaired due to TCR defects, RNA polymerase II is stalled, which triggers apoptosis [23]. The mechanism of platinum-based chemotherapy is the formation of platinum-DNA adducts followed by intra- and inter-strand crosslinks, which inhibit DNA replication and lead to apoptosis [24]. Since TCR deficiency and platinum-based chemotherapy both involve gene disruption, synthetic lethality can occur if both are present at the same time [7]. Therefore, TCR deficiencies will enhance the response to platinum chemotherapy as demonstrated in an experimental study using human cells [25]. In addition, as GGR deficiency in the NER pathway did not correlate with the responsiveness of platinum chemotherapy in that study, it appears that TCR deficiency in the NER pathway is more associated with responsiveness to platinum chemotherapy [25]. Consistent with this, our study revealed that patients had a better response to the FOLFIRINOX regimen, which contains the platinum-based anticancer drug oxaliplatin, when they carried a variant in *ERCC6*, a major component of TCR. Platinum-based chemotherapy has been widely used in various cancers besides pancreatic cancer, and there have been several genetic studies related to platinum-based chemotherapy responses [26,27,28]. Among those studies, Cui et al. reported that *ERCC6* is associated with platinum-based chemotherapy responses in lung cancer patients [29]. The genetic variant of *ERCC6* reported in that study is also rs2228528, consistent with our study [29]. There were several previous studies investigating genes associated with platinum-based chemotherapy responses, including FOLFIRINOX in pancreatic cancer [11]. These previous studies reported that patients with mutations in the DDR gene showed a good response to FOLFIRINOX, but none of the genes associated with that response were related to the NER pathway [11,12]. Most previous studies were conducted using targeted gene sequencing that did not probe for *ERCC6*, so the gene in the NER pathway related to platinum-based chemotherapy response may not have been found [11,12,30]. However, by analyzing 148 DDR genes based on whole exome sequencing, we were able to find a novel genetic variant of *ERCC6* that is associated with FOLFIRINOX responses. There were no human data on chemotherapy agents related to *ERCC6* other than platinum chemotherapy agents, and there was one report showing that the anticancer effect of 5-fluorouracil was significantly increased when *ERCC6* was knocked down in colorectal cancer cell lines and xenograft models [31]. Although the above study did not use pancreatic cancer cell, considering the inclusion of 5-fluorouracil in FOLFIRINOX regimen, it is likely that 5-fluorouracil in addition to oxaliplatin may have influenced the difference in response to FOLFIRINOX according to the *ERCC6* variant. This should be confirmed through future studies using pancreatic cancer cells.

To date, the most well-known DDR gene mutation related to treatment responsiveness to FOLFIRINOX in pancreatic cancer is the BRCA mutation [8]. The percentage of pathogenic BRCA mutations was as small as 1% in our study, and statistical significance may not have been found with respect to the response to FOLFIRINOX due to the small number of patients. Although there are differences according to reports, the prevalence of germline BRCA mutations in patients with pancreatic cancer is known to be highest at 10–14% in patients with Ashkenazi Jewish ancestry, and 4–7% in other Western patients [9,32]. In Asian patients, there are no reports based on a large cohort, but a study reported by Lee et al. showed a relatively low prevalence (0.6%) of germline BRCA mutations, which is consistent with our study [10]. Compared to the relatively low frequency of BRCA mutations, rs2228528 in *ERCC6* found in our study showed a high variant allele frequency (VAF) of 42.2%. According to large-scale reference genomic data, rs2228528 in *ERCC6* is a germline variant with a VAF of 18–24% globally and 41–47% in East Asia, showing similar frequency to our study [33,34,35]. Considering this high VAF, rs2228528 in *ERCC6* is a good candidate for a biomarker to predict FOLFIRINOX responses, and is expected to be an especially useful biomarker among Asian pancreatic cancer patients with low BRCA mutation frequency. Additionally, rs2228528 found in this study is a germline variant showing a VAF of around 40% and can be easily checked using blood samples. Therefore, if future studies validate the association between rs2228528 in *ERCC6* and response to FOLFIRINOX, using blood samples, a single blood draw could provide an easy and fast way to determine whether to use FOLFIRINOX treatment or gemcitabine-based chemotherapy first in pancreatic cancer patients. If rs2228528 in *ERCC6* has a significant effect on protein expression, screening for this SNP using an immunohistochemistry-based technique may be possible, but this has not been revealed yet. In addition, since the cost of genetic testing has recently become cheaper and rs2228528 in *ERCC6* may be confirmed through a single blood draw, screening through this will be more accurate and efficient. Furthermore, poly(adenosine diphosphate-ribose) polymerase (PARP) inhibitors are also known to elicit a good response in pancreatic cancer patients with DDR gene mutations, so future studies are needed to determine whether *ERCC6* mutations play a role in the response to PARP inhibitors [36].

It is unclear whether rs2228528 in *ERCC6* directly affects the progression of pancreatic cancer regardless of chemotherapy. However, among DNA damage repair genes such as *ERCC6*, the well-known BRCA gene itself is considered to have no significant effect on the progression of pancreatic cancer, and it seems to show a difference in survival through a difference in responsiveness to platinum-based chemotherapy [37]. Similarly, it can be assumed that the rs2228528 in *ERCC6* itself does not affect the progression of pancreatic cancer.

This study had several strengths. First, this is the largest study to date on genes related to the responsiveness of FOLFIRINOX chemotherapy in patients with pancreatic cancer. Second, this study discovered novel *ERCC6* variants by conducting genetic analysis based on whole exome sequencing rather than using the targeted gene sequencing methods from previous studies.

There are some limitations in this study. First, this study was conducted in a single institution and analyzed in a retrospective manner, although a prospective database was used. This retrospective nature may have caused selection bias. In particular, in order to include only patients whose PFS was measured more accurately, we excluded patients with follow-up loss or patients who could not continue chemotherapy due to poor performance. As a result, selection bias may have occurred in the direction of including patients with better performance status. However, it would be difficult for this direction of selection bias to work solely to make the insignificant *ERCC6* variant appear significantly, and rather, it is meaningful that even in patients with good performance, the responsiveness to FOLFIRINOX was different according to the *ERCC6* variant. Second, there was no validation process in this study. Whether rs2228528 alters protein structure has yet to be confirmed, so further research is needed on this, and validation studies in large-scale patients are required. Third, there was no matched blood sample that could identify the germline variant, so the distinction between germline and somatic variants was not made. However, this distinction could be roughly made with existing large-scale genomic data, and in the case of DDR gene mutations, both germline and somatic mutations are known to have an effect on the response to platinum-based chemotherapy. Therefore, this distinction of germline and somatic mutation should not have a critical effect on the results of this study [11,38].

## 5. Conclusions

In conclusion, we found a novel variant in *ERCC6* associated with improved PFS in pancreatic cancer patients who underwent FOLFIRINOX chemotherapy. If validated through future large-scale studies, rs2228528 in *ERCC6* could be used as a valuable biomarker to help determine whether to use FOLFIRINOX as the first-line therapy in pancreatic cancer patients.

## Figures and Tables

**Table 1:** Patient characteristics.

Variables	Median (IQR) or Number (%)
Age, years	64.0 (58.0–70.0)
Sex	
Male	58 (56.3)
Female	45 (43.7)
Body mass index, kg/m^2^	22.9 (21.1–25.1)
ECOG performance status	
0	99 (96.1)
1	4 (3.9)
Tumor location	
Head	43 (41.7)
Body/tail	60 (58.3)
Clinical T stage	
T1–3	38 (36.9)
T4	65 (63.1)
Clinical N stage	
N0	73 (70.9)
N1–2	30 (29.1)
Clinical M stage	
M0	66 (64.1)
M1	37 (35.9)
Serum CA 19-9, U/mL	675.0 (63.0–4492.0)
Best response to FOLFIRINOX chemotherapy	
Partial response	33 (32.0)
Stable disease	57 (55.3)
Progressive disease	13 (12.6)
Resection after FOLFIRINOX chemotherapy	41 (39.8)

Abbreviations: IQR, interquartile range; ECOG, Eastern Cooperative Oncology Group; CA 19-9, carbohydrate antigen 19-9.

**Table 2:** Multivariate Cox proportional hazard regression of factors associated with progression-free survival (PFS).

Variables	HR (95% CI)	*p*
*ERCC6* genotype	0.539 (0.371–0.783)	0.001
GG	reference	
GA		
AA		
Age	1.032 (0.998–1.066)	0.062
Sex	0.757 (0.449–1.275)	0.295
Male	reference	
Female		
T stage	0.691 (0.382–1.251)	0.223
T1–3	reference	
T4		
N Stage	1.574 (0.893–2.774)	0.116
N0	reference	
N1–2		
M Stage	2.209 (1.159–4.209)	0.016
M0	reference	
M1		
Tumor location	0.578 (0.311–1.075)	0.083
Head	reference	
Body/tail		
Body mass index	0.860 (0.500–1.481)	0.587
≤23	reference	
>23		
CA 19-9	0.915 (0.491–1.706)	0.780
≤37	reference	
>37		
Surgical resection after FOLFIRINOX	0.265 (0.135–0.520)	<0.001
No	reference	
Yes		

Abbreviations: HR, hazard ratio; CI, confidence interval; CA 19-9, carbohydrate antigen 19-9.

## Data Availability

The data presented in this study are available on request from the corresponding author.

## Supplementary Materials

The following are available online at https://www.mdpi.com/2072-6694/13/6/1196/s1, Table S1: Detailed list of DNA damage repair genes.
