# Supplementary material for: Excision Repair Cross-Complementation Group 6 Gene Polymorphism Is Associated with the Response to FOLFIRINOX Chemotherapy in Asian Patients with Pancreatic Cancer
Source: Cancers (Basel). 2021 Mar 10;13(6):1196. doi: 10.3390/cancers13061196 (PMC7998301; doi:10.3390/cancers13061196)
Supplement: Supplementary file 1 [file cancers-13-01196-s001.pdf]

cancers MDPI

Supplementary Material: Excision Repair Cross-Complementation Group 6 Gene Polymorphism is Associated with the Response to FOLFIRINOX Chemotherapy in Asian Patients with Pancreatic Cancer

Young Hoon Choi [1,2,†], Younggyun Lim [3,†], Ji Kon Ryu [2,*], Woo Hyun Paik [2], Sang Hyub Lee [2], Yong-Tae Kim [2] and Ju Han Kim [3]

Table 1. Detailed list of DNA damage repair genes.

DNA damage repair pathway	Genes
Base excision repair	APEX1, APEX2, APLF, DUT, LIG3, MBD4, MPG, MUTYH, NEIL1, NEIL2, NEIL3, NTHL1, NUDT1, OGG1, PARP1, PARP2, PNKP, POLB, RECQL4, SMUG1, TDG, TDP1, UNG, WRN, XRCC1
Direct reversal repair	ALKBH2, ALKBH3, MGMT
DNA damage response	ATM, ATR, ATRIP, CHEK1, CHEK2, CLK2, CLSPN, H2AFX, HUS1, MDC1, NABP2, PER1, PER2, RAD1, RAD17, RAD9A, RNF8, TOP2A, TOPBP1, TP53, TP53BP1, UBE2N
Fanconi Anemia	BRCA2, BRIP1, FAAP100, FAAP24, FANCA, FANCB, FANCC, FANCD2, FANCE, FANCF, FANCG, FANCI, FANCL, FANCM, PALB2, RAD51C, SLX4, USP1, WDR48
Homologous recombination	BARD1, BLM, BRCA1, DMC1, EME1, EME2, GEN1, HELQ, MRE11A, MUS81, NBN, RAD50, RAD51, RAD51B, RAD51D, RAD52, RAD54B, RAD54L, RBBP8, RECQL, RECQL5, SHFM1, SLX1A, SLX1B, XRCC2, XRCC3
Mismatch repair	MLH1, MLH3, MSH2, MSH3, MSH4, MSH5, MSH6, PCNA, PMS1, PMS2, POLD1, POLE
Non-homologous end joining	DCLRE1C, LIG4, NHEJ1, POLL, POLM, PRKDC, PRPF19, SETMAR, XRCC4, XRCC5, XRCC6
Nucleotide excision repair	CCNH, CDK7, CETN2, DDB1, DDB2, ERCC1, ERCC2, ERCC3, ERCC4, ERCC5, ERCC6, ERCC8, GTF2H1, GTF2H2, GTF2H3, GTF2H4, GTF2H5, LIG1, MMS19, MNAT1, RAD23A, RAD23B, RPA1, RPA2, RPA3, RPA4, UVSSA, XAB2, XPA, XPC
